# Detection of pathogens associated with early-onset neonatal sepsis in cord blood at birth using quantitative PCR

**DOI:** 10.12688/wellcomeopenres.17386.1

**Published:** 2022-01-05

**Authors:** Christina W. Obiero, Wilson Gumbi, Stella Mwakio, Hope Mwangudzah, Anna C. Seale, Mami Taniuchi, Jie Liu, Eric Houpt, James A. Berkley

**Affiliations:** 1 Clinical research, KEMRI-Wellcome Trust Research Programme, Kilifi, Kenya; 2 Global health, Academic Medical Center of the University of Amsterdam, Amsterdam, The Netherlands; 3 Bioscience department, KEMRI-Wellcome Trust Research Programme, Kilifi, Kenya; 4 Epidemiology and Population Health, London School of Hygiene & Tropical Medicine, London, UK; 5 College of Health and Medical Sciences, Haramaya University, Harar, Ethiopia; 6 Division of Infectious Diseases and International Health, University of Virginia, Virginia, USA; 7 Centre for Tropical Medicine, University of Oxford, Oxford, UK; 8 The Childhood Acute Illness & Nutrition (CHAIN) Network, Nairobi, Kenya

**Keywords:** Neonate, sepsis, molecular, aetiology, PCR

## Abstract

**Background: **Early onset neonatal sepsis (EONS) typically begins prior to, during or soon after birth and may be rapidly fatal. There is paucity of data on the aetiology of EONS in sub-Saharan Africa due to limited diagnostic capacity in this region, despite the associated significant mortality and long-term neurological impairment.

**Methods: **We compared pathogens detected in cord blood samples between neonates admitted to hospital with possible serious bacterial infection (pSBI) in the first 48 hours of life (cases) and neonates remaining well (controls). Cord blood was systematically collected at Kilifi County Hospital (KCH) from 2011-2016, and later tested for 21 bacterial, viral and protozoal targets using multiplex PCR via TaqMan Array Cards (TAC).

**Results: **Among 603 cases (101 [17%] of whom died), 179 (30%) tested positive for ≥1 target and 37 (6.1%) tested positive for multiple targets. *Klebsiella oxytoca*, *Escherichia coli/Shigella* spp., *Pseudomonas aeruginosa,* and *Streptococcus pyogenes* were commonest. Among 300 controls, 79 (26%) tested positive for ≥1 target, 11 (3.7%) were positive for multiple targets, and *K. oxytoca* and *P. aeruginosa *were most common. Cumulative odds ratios across controls: cases (survived): cases (died) were *E. coli/Shigella* spp. 2.6 (95%CI 1.6-4.4); *E. faecalis* 4.0 (95%CI 1.1-15); *S. agalactiae* 4.5 (95%CI 1.6-13); *Ureaplasma* spp. 2.9 (95%CI 1.3-6.4); Enterovirus 9.1 (95%CI 2.3-37); and *Plasmodium* spp. 2.9 (95%CI 1.4-6.2). Excluding *K. oxytoca* and *P. aeruginosa* as likely contaminants, aetiology was attributed in 9.4% (95%CI 5.1-13) cases using TAC. Leading pathogen attributions by TAC were *E. coli/Shigella* spp. (3.5% (95%CI 1.7-5.3)) and *Ureaplasma* spp. (1.7% (95%CI 0.5-3.0)).

**Conclusions: **Cord blood sample may be useful in describing EONS pathogens at birth, but more specific tests are needed for individual diagnosis. Careful sampling of cord blood using aseptic techniques is crucial to minimize contamination. In addition to culturable bacteria, *Ureaplasma* and Enterovirus were causes of EONS.

## Introduction

Forty-one percent of global neonatal deaths occur in sub-Saharan Africa ^1^ and the risk of dying is highest in the first week of life ^2^. Infection is a leading cause of neonatal mortality, accounting for ~37% of deaths in sub-Saharan Africa ^3^, and is associated with long-term neurological impairment ^4^. Early-onset neonatal sepsis (EONS) is often due to maternal transmission of pathogens ^5^ prior to, during, or soon after birth, and can be rapidly fatal. Neonatal sepsis lacks a consensus definition and the reference point for EONS is variable, based on the timing of onset of symptoms or sampling of a positive culture ^6^, i.e., occurring within the first 48 hours ^7, 8^, 72 hours ^9^ or seven days ^10^ of life. Most research conducted in developing countries has focused on culturable bacterial pathogens, with *Klebsiella* spp. *, Escherichia coli* and *Staphylococcus aureus* identified as leading causes of EONS ^11, 12^. Group B Streptococcus (GBS) has been variably implicated in EONS, and may be underestimated due to its rapid fatality and surveillance methodology ^13– 15^. There are limited published data on viruses such as Herpes Simplex Virus (HSV) ^16^ and Cytomegalovirus (CMV) ^17^ as causes of EONS in this setting.

Blood culture is the gold standard diagnostic test for EONS, despite low sensitivity ^18, 19^. One to two millilitres of blood volume is recommended to improve microorganism recovery ^20^, but smaller volumes are often obtained from sick neonates. Intrapartum antimicrobials ^21^, presence of fastidious organisms and culture contamination may also contribute to low culture yields. Lack of availability of microbiology facilities, lengthy turn-around times ^22^ and high rates of culture-negative sepsis contribute to antibiotic consumption ^19^, exacerbating antimicrobial resistance ^23^, affecting the gut microbiota ^24^, and potentially missing important non-culturable organisms.

Nucleic acid amplification techniques can detect a broad range of pathogens ^25^ with up to 90% sensitivity and 93% specificity compared to microbial culture in some studies ^22^. Recently, a custom TaqMan Array Card (TAC) approach based on quantitative reverse-transcription polymerase chain reaction (RT-qPCR) ^26^ was applied to neonatal blood and respiratory samples in South Asia and South Africa ^27, 28^. Causal attribution of organisms identified in blood and respiratory samples in EONS using latent class modelling was 23% in South Asia ^28^ and 27% in South Africa ^27^. Bacteria were predominant and *Ureaplasma* spp. was identified as a significant pathogen in these studies. However, healthy controls were not sampled in identical circumstances to cases in South Asia (cases were recruited from study health facilities while controls were identified from the community using an automated algorithm; controls were older than cases at sample collection) ^28^ whilst in South Africa both cases and controls were recruited from the study hospital ^27^.

Cord blood provides a potential opportunity for early pathogen detection prior to the clinical onset of infection, and with adequate sample volumes ^29^. Biomarkers in cord blood may correlate with peripheral blood parameters including total and differential white blood cell counts ^30^, and acute phase reactants such as C-reactive protein, serum amyloid A, haptoglobin ^31^, interleukin-6 and procalcitonin ^32^. Culture, PCR and sequencing have identified pathogenic bacteria and correlate with acute phase reactants in cord blood ^33, 34^. However, cord blood contamination may easily occur ^29, 33, 35^.

We hypothesized that pathogens detected in cord blood using a molecular technique would be associated with subsequent admission and death with suspected EONS. In a nested case control study of cord blood samples systematically collected at birth, we selected neonates hospitalized within 48 hours of life with possible serious bacterial infection (pSBI) and a random set of neonates who were sampled identically and remained well.

## Materials and methods

### Study design and participants

We performed a retrospective case-control study of cord blood samples obtained at delivery at Kilifi County Hospital (KCH) within a systematic clinical surveillance of maternal and neonatal adverse events (clinicaltrials.gov NCT01757028) ^36^. KCH serves a mostly rural population along the Kenyan coast. About half of all admissions to the neonatal ward are from the KCH maternity department, where there are ~4000 deliveries per year. Hospital deliveries and neonatal admissions have increased since maternity user fees were abolished (Free Maternity Service policy, 2013) ^37^. Maternal clinical data and cord blood samples were obtained and analysed during the surveillance (clinicaltrials.gov NCT01757028) ^36^ and stored for future research. Data were collected using a standardized maternal admission record ^36^. Cord blood samples were obtained by trained clinicians using standard aseptic techniques and universal safety precautions. After delivery of the neonate and the placenta, the umbilical cord was swabbed using 70% isopropyl alcohol and spirit, double clamped and cut. Approximately 10 ml of venous cord blood was collected using either a sterile 18-gauge needle (preferred) or 5Fr gastric tube and a syringe into ethylenediamine tetra-acetic acid (EDTA) tubes (BD Diagnostics, USA), centrifuged within an hour of collection; plasma and cell pellet aliquots were then frozen separately at -80°C.

**Figure 1.**
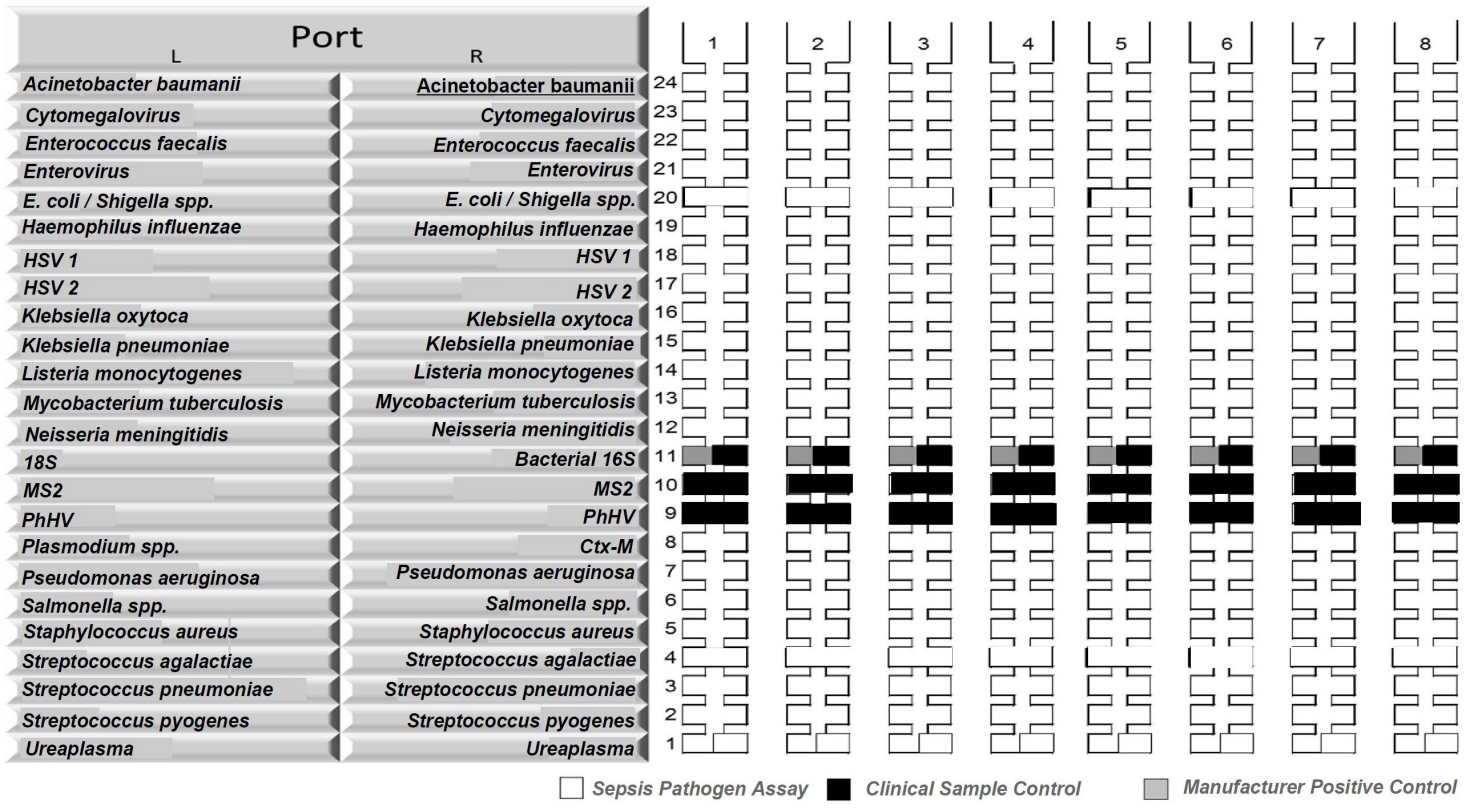
Organisms included in the whole blood TaqMann Array Card panel. Pathogens interrogated with TaqMan Array Card (TAC) in whole blood. The TAC is a 384-well real-time quantitative reverse transcription polymerase chain reaction (RT-qPCR) based platform consisting of 8 (shown as no. 1 to 8 above) individual microfluidic channels that can be loaded with PCR reactions containing nucleic acid extract from a clinical specimen or control material. The TAC was customised to include 21 targets (16 bacterial, 4 viral and 1 protozoal organism, tested in duplicate) and two controls (MS2 bacteriophage and phocine Herpesvirus (PhHV)). The 21 targets are shown in alphabetical order as follows: *Acinetobacter baumanii*, Cytomegalovirus, *Enterococcus faecalis*, Enterovirus, *Escherichia coli*/ *Shigella* spp., *Haemophilus influenzae*, Herpes Simplex Virus 1, Herpes Simplex Virus 2, *Klebsiella oxytoca*, *Klebsiella pneumoniae*, *Listeria monocytogenes*, *Mycobacterium tuberculosis*, *Neisseria meningitidis*, *Plasmodium* spp., *Pseudomonas aeruginosa*, *Salmonella enterica*, *Staphylococcus aureus*, *Streptococcus agalactiae*, *Streptococcus pneumoniae*, *Streptococcus pyogenes*, and *Ureaplasma* spp.

Neonates born between March 2011 and March 2016 ( Figure 1) who were resident of the Kilifi Health and Demographic Surveillance System (KHDSS) ^38^ and had cord blood samples available were considered for this analysis. Cases were defined as neonates hospitalized within 48 hours of life with one or more features of possible serious bacterial infection (pSBI): history of difficulty feeding, history of convulsions, movement only when stimulated, respiratory rate of ≥60 breaths/min, severe chest indrawing, and a temperature of ≥37.5°C, or ≤35.5°C ^39^. Cases were further categorized as those who died during hospitalization, and those who survived and discharged home well. Unmatched controls were randomly drawn from neonates who had cord blood samples taken in identical circumstances, did not have pSBI, were discharged home well after delivery, and survived for at least 60 days without hospitalization, determined using the KHDSS household census.

Clinical data for hospitalised neonates was systematically collected at admission using standard proforma and entered in real-time on a database within a surveillance for invasive bacterial disease ^38, 40^. Routine laboratory investigations for clinical care included blood culture (BACTEC Peds Plus/F bottles and BACTEC 9050 instrument, Becton Dickinson, UK) and cerebrospinal fluid (CSF) culture where indicated, as previously described ^41^. *Bacillus* spp., Coagulase-negative *Staphylococci*, Coryneforms, *Micrococcus* spp., and viridans *Streptococci* were considered clinically non-significant blood culture isolates in this context.

The Kenya Medical Research Institute Scientific Steering Committee (KEMRI SSC) approved collection of cord blood samples and clinical data (SSC 1778 and 1433). Written informed consent for collection and use of samples and data for research was obtained from all participants’ parents/guardians. This retrospective analysis was approved by the KEMRI Scientific Ethics Review Unit (SERU 3007). All studies were conducted according to relevant guidelines and regulations.

### Study size

We estimated that 600 pSBI cases and 300 controls would give 90% power and 5% alpha for minimum proportions pathogens detected in 5% of pSBI cases and 1% in healthy controls.

### Total nucleic acid extraction 

Stored cord blood plasma and pellets were mixed after thawing and total nucleic acid (TNA) was extracted using the High Pure viral nucleic acid large volume kit (Roche 05114403001) following manufacturer’s instructions. For each experiment, up to 2.5 ml of the plasma/pellets mix (1.5:1) underwent a lysate preparation process, then purification and elution of 150 µl TNA using spin columns, including a High Pure Extender Assembly for large initial volume. Extrinsic controls, Phocine Herpesvirus (PhHV) and artificial construct containing the region targeted by the MS2 PCR assay were added to each sample during lysate preparation to evaluate extraction and amplification efficiency. For each batch of extractions, a blank (about 2.5 ml of nuclease-free water) was processed through the complete protocol, and later assayed to rule out contamination during the extraction and amplification processes. A positive target in the blank would invalidate positive results for that target in the same batch of TNA extractions. Testing of TNA on TAC was done either on the same day or the day following extraction.

### Detection of targets using TAC RT-qPCR

Real-time reverse transcription PCR assays were performed using a custom TAC (Thermo Fisher, CA, USA) on a QuantStudio 7 Flex instrument (Life Technologies, USA) to detect 16 bacterial, four viral and one protozoal targets ( Figure 1) ^42, 43^. The choice of targets was based on previous studies on neonatal sepsis ^28^. The uidA gene detects both *E. coli* and *Shigella* species, hence were included as a single target on the TAC cards. Primers and probes were adapted from published assays to detect acute febrile illness ^42, 44^ and sepsis ^43^ optimized for the universal cycling conditions on the card. Positive controls were plasmids for DNA and *in vitro* transcripts for RNA. Cards were designed, quality-controlled, and validated at the University of Virginia who provided onsite training.

For each experiment, 25 µL of TaqMan Fast Virus one-step master mix (4444434, Applied Biosystems, Thermo Fisher Scientific) was mixed with 75 µL of TNA extract or nuclease-free water (for no template control [NTC]) to make a 100 µL PCR reaction mix. Each 100 µL PCR reaction + sample mix was then transferred into the fill port of TAC after which the TAC was then centrifuged to ensure complete filling of the reaction wells, sealed and run. The reactions included a reverse transcription at 50°C for 10 minutes, initial denaturation at 95°C for 20 seconds, then 40 three-second cycles of 95°C , and 60°C for 30 seconds. Up to eight samples were tested per card, blinded to case-control status, with one NTC included in every 10 cards to check for reagent contamination. Analysis utilized QuantStudio Real-Time PCR Software version 1.2 (Applied Biosystems, Thermo Fisher). Results were quality-checked by examining target amplification plots. Baseline adjustment for targets or reaction wells with irregular amplification was done when a false amplification curve was generated or an inaccurate threshold cycle (Ct) value was yielded. Upon review of the reaction fluorescence curves for each target, we set the cut off threshold cycle (Ct) value for all targets at <40. Samples were deemed positive when any of the duplicate reactions yielded amplification curves that crossed the threshold as defined and controls were valid. We repeated *Ureaplasma* spp. testing using singleplex PCR on 261 samples which had parallel positive blanks on TAC, and excluded TAC results from four samples for which repeat singleplex PCR was not possible due to depletion of TNA.

### Statistical analysis

Characteristics associated with case status were investigated using backward stepwise logistic regression retaining variables with P<0.1. We initially estimated the odds ratio for all cases (survived and died) versus controls. Then, since several organisms of potential public health relevance were not detected at all in controls and could not be meaningfully analysed in this way, we estimated the cumulative odds of pSBI across ordered groups of controls: cases-survived: cases-died using ordinal logistic regression which can accommodate zero values. We tested the proportional odds assumption to confirm that the relationship between each pair of outcome variable (controls, cases-survived, and cases-died) were similar prior to performing ordinal logistic regression. We estimated the attribution fraction (AF) among cases with “punafcc” in STATA v15 (StataCorp, TX, USA) ^45^.

## Results

**Figure 2.**
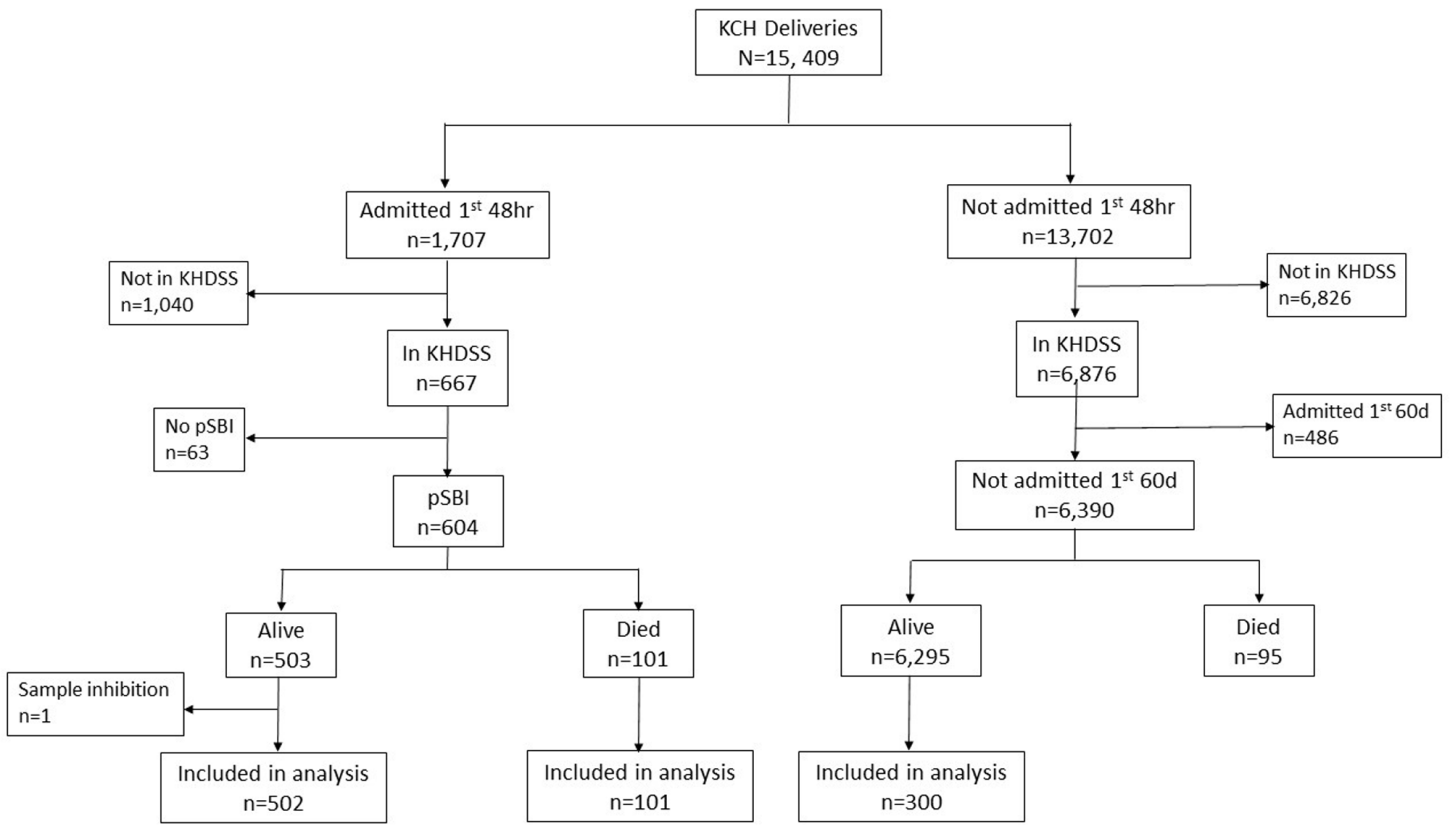
Study Participant Flow. Selection of cases and controls from a cohort of 15,409 deliveries at Kilifi County Hospital (KCH) between March 2011 and March 2016. Cases were hospitalised within the first 48 hours of life, resident of the Kilifi Health Demographic Surveillance System (KHDSS) and presented with one or more of the WHO-defined criteria for possible serious bacterial infection (pSBI). Controls were resident of the KHDSS and not hospitalised within the first 60 days of life.

Of 15,409 deliveries during the study period, 604 cases and 300 controls were selected ( Figure 2). One case was subsequently excluded due to sample inhibition to amplification. Thus, 603 cases comprising 502 EONS survivors and 101 EONS deaths (58 [57%] and 74 [73%] of whom died within 24 and 48 hours after birth respectively) were included. Admissions on day 0, 1 and 2 of life among EONS cases were as follows: 256 (51%), 184 (37%) and 62 (12%) respectively in 502 survivors, compared with 93 (92%), 7 (6.9%) and 1 (1.0%) in 101 deaths (P<0.001).

Compared with controls, pSBI cases were more likely to be born of mothers who were nulliparous (odds ratio [OR] 1.7, 95% confidence interval [CI] 1.2-2.3) or presented with drainage of liquor (OR 2.0, 95% CI 1.3-3.1), vaginal bleeding (OR 4.8, 95% CI 2.1-11) or oedema (OR 3.0, 95% CI 1.3-6.9) ( Table 1). Admission with pSBI was not associated with maternal fever, prolonged rupture of membranes (PROM) or abnormal urinalysis at admission (prior to delivery).

**Table 1.** Maternal characteristics.

Characteristic	Cases (n=603)	Controls (n=300)	Univariable Odds Ratio (95% CI) ^a^	P value ^a^	Multivariable Odds Ratio (95% CI) ^b^	P value ^b^
Age, years	26 (21-32)	26 (21-32)	1.0 (0.9 to 1.0)	0.593	-	-
Weight, kg	60 (53-70)	60 (54-67)	1.0 (0.9 to 1.0)	0.632	-	-
Height, cm	156 (151-160)	156 (151-160)	0.9 (0.9 to 1.0)	0.283	-	-
MUAC, cm	26 (24-28)	25 (24-28)	1.0 (0.9 to 1.1)	0.326	-	-
Marital status						
Married	543 (90)	264 (88)	1.0	-	-	-
Single	38 (6.3)	24 (8.0)	0.8 (0.5 to 1.3)	0.335	-	-
Divorced	2 (0.3)	2 (0.7)	0.5 (0.1 to 3.5)	0.472	-	-
Widowed	3 (0.5)	1 (0.3)	1.5 (0.2 to 14)	0.744	-	-
Missing	17 (2.8)	9 (3.0)	0.9 (0.4 to 2.1)	0.839	-	-
Education Level						
None	105 (17)	58 (19)	1.0	-	-	-
Primary	360 (60)	178 (59)	1.1 (0.8 to 1.6)	0.555	-	-
Secondary	86 (14)	36 (12)	1.3 (0.8 to 2.2)	0.281	-	-
Higher	34 (5.6)	12 (4.0)	1.6 (0.8 to 3.3)	0.230	-	-
Missing	18 (3.0)	16 (5.3)	0.6 (0.3 to 1.3)	0.211	-	-
Nulliparous						
No	383 (63)	207 (69)	1.0	-	1.0	-
Yes	185 (31)	65 (22)	1.5 (1.1 to 2.1)	0.010	1.7 (1.2 to 2.3)	0.004
Missing	35 (6.0)	28 (9.0)	0.7 (0.4 to 1.1)	0.143	0.7 (0.4 to 1.3)	0.304
*Presenting complaints*						
History of fever						
No	584 (97)	294 (98)	1.0	-	-	-
Yes	10 (1.7)	1 (0.3)	5.0 (0.6 to 40)	0.124	-	-
Missing	9 (1.5)	5 (1.7)	0.9 (0.3 to 2.7)	0.861	-	-
Drainage of liquor						
No	489 (81)	266 (89)	1.0	-	1.0	-
Yes	109 (18)	30 (10)	2.0 (1.3 to 3.0)	0.002	2.0 (1.3 to 3.1)	0.002
Missing	5 (0.8)	4 (1.3)	0.7 (0.2 to 2.6)	0.568	0.0 (0.0 to 0.0)	0.975
Ruptured membranes						
No	401 (66)	210 (70)	1.0	-	-	-
Yes	172 (29)	77 (26)	1.2 (0.9 to 1.6)	0.331	-	-
Missing	30 (5.0)	13 (4.3)	1.2 (0.6 to 2.4)	0.581	-	-
PROM >18h						
No	471 (78)	240 (80)	1.0	-	-	-
Yes	27 (4.5)	9 (3.0)	1.5 (0.7 to 3.3)	0.280	-	-
Missing	105 (17)	51 (17)	1.0 (0.7 to 1.5)	0.799	-	-
Vaginal bleeding						
No	539 (89)	287 (96)	1.0	-	1.0	-
Yes	61 (10)	7 (2.3)	4.6 (2.1 to 10)	<0.001	4.8 (2.1 to 11)	<0.001
Missing	3 (1.0)	6 (2.0)	0.3 (0.1 to 1.1)	0.063	0.0 (0.0 to 0.0)	0.993
Dysuria						
No	577 (96)	287 (96)	1.0	-	-	-
Yes	21 (3.5)	8 (2.7)	1.3 (0.6 to 3.0)	0.527	-	-
Missing	5 (0.8)	5 (1.7)	0.5 (0.1 to 1.7)	0.273	-	-
Decreased foetal movements						
No	576 (96)	293 (98)	1.0	-	1.0	-
Yes	23 (3.8)	1 (0.3)	11.7 (1.6 to 87)	0.016	6.0 (0.8 to 47)	0.085
Missing	4 (0.7)	6 (2.0)	0.3 (0.1 to 1.2)	0.096	0.0 (0.0 to 0.0)	0.993
*Admission examination*						
Emergency signs ^c^						
No	500 (83)	271 (90)	1.0	-		
Yes	98 (16)	27 (9.0)	2.0 (1.3 to 3.1)	0.003	1.6 (1.0 to 2.5)	0.058
Missing	5 (0.8)	2 (0.7)	1.4 (0.3 to 7.0)	0.718	0.0 (0.0 to 0.0)	0.976
Temperature, °C						
36-38	483 (80)	252 (84)	1.0	-	-	-
>38	7 (1.2)	0 (0.0)	1.0	-	-	-
<36	47 (7.8)	17 (5.7)	1.4 (0.8 to 2.6)	0.212	-	-
Missing	66 (11)	31 (10.3)	1.1 (0.7 to 1.7)	0.649	-	-
Oedema						
No	551 (91)	287 (96)	1.0	-	1.0	-
Yes	48 (8.0)	7 (2.3)	3.6 (1.6 to 8.0)	0.002	3.0 (1.3 to 6.9)	0.009
Missing	4 (0.7)	6 (2.0)	0.3 (0.1 to 1.2)	0.103	1.0	-
Positive nitrite and/or leucocytes (2+/3+) ^d^						
No	417 (69)	214 (71)	1.0	-	-	-
Yes	108 (18)	46 (15)	1.2 (0.8 to 1.8)	0.339	-	-
Missing	78 (13)	40 (13)	1.0 (0.7 to 1.5)	0.997	-	-
Antibiotics in the last 4 weeks						
No	539 (89)	274 (91)	1.0	-	-	-
Yes	61 (10)	26 (8.7)	1.2 (0.7 to 1.9)	0.473	-	-
Missing	3 (1)	0 (0)	1.0	-	-	-

Data are N (%) or median (IQR) Abbreviations: CI, confidence interval; kg, kilogram; cm, centimetre; MUAC, mid-upper arm circumference; PROM, prolonged rapture of membranes; °C, degree Celsius. ^a^Univariable logistic model for all cases vs. controls ^b^Multivariable logistic model for all cases vs. controls, including variables with P&0.1 ^c^Danger signs at triage suggesting need for emergency care (include airway not patent, respiratory rate >30 or <10 breaths/minute, systolic blood pressure >160 or <90 mmHg, diastolic blood pressure >90 mmHg, heart rate <40 or >120 beats/minute, unconscious or alert only to pain, other obstetric emergencies (including imminent delivery) requiring immediate intervention) ^d^Urinalysis results at admission

Among newborns, assessment of appearance, pulse, grimace, activity, and respiration (APGAR) score <9 at 5 minutes (OR 15, 95% CI 6.2-35), resuscitation at birth (OR 3.6, 95% CI 1.8-7.3) and gestation of <32 weeks (OR 2.9, 95% CI 1.1-7.7) were associated with a pSBI case status ( Table 2). Newborn mid-upper arm circumference (MUAC) (OR 0.77, 95% CI 0.64-0.94 per cm) and head circumference (OR 0.90, 95% CI 0.81-0.99 per cm) were also associated with pSBI, but birth weight was not associated with pSBI (OR 1.2, 95% CI 0.73-2.0) in this adjusted model.

**Table 2.** Neonatal birth characteristics.

Characteristic	Cases (n=603)	Controls (n=300)	Univariable Odds Ratios (95% CI) ^a^	P value ^a^	Multivariable Odds Ratios (95% CI) ^b^	P value ^b^
Weight, kg	2.7 (1.9-3.2)	3 (2.7-3.3)	0.4 (0.3 to 0.5)	<0.001	1.2 (0.7 to 2.0)	0.454
Length, cm	47.5 (43.0-49.5)	48.5 (47.0-50.0)	0.9 (0.8 to 0.9)	<0.001	1.0 (0.9 to 1.1)	0.814
MUAC, cm	10.0 (8.2-10.7)	10.5 (9.8-11.2)	0.6 (0.5 to 0.7)	<0.001	0.8 (0.6 to 0.9)	0.010
Head circumference, cm	33.3 (31.0-34.9)	34.0 (33.0-35.0)	0.8 (0.8 to 0.9)	<0.001	0.9 (0.8 to 0.9)	0.036
Sex						
Male	345 (57)	151 (50)	1.0	-	1.0	-
Female	258 (43)	149 (50)	0.8 (0.6 to 1.0)	0.051	0.8 (0.6 to 1.1)	0.177
Gestation, weeks						
≥37	373 (62)	247 (82)	1.0	-	1.0	-
≥32 to <37	122 (20)	44 (15)	1.8 (1.3 to 2.7)	0.002	1.2 (0.8 to 1.9)	0.457
<32	88 (15)	6 (2.0)	9.7 (4.2 to 23)	<0.001	2.9 (1.1 to 7.7)	0.035
Missing	20 (3.3)	3 (1.0)	4.4 (1.3 to 15)	0.017	3.0 (0.8 to 11)	0.096
Mode of delivery						
Vaginal	441 (73)	205 (68)	1.0	-	-	-
Caesarean section	162 (27)	91 (30)	0.8 (0.6 to 1.1)	0.225	-	-
Missing	0 (0.0)	4 (1.3)	1.0	-	-	-
Resuscitated at birth ^c^						
No	415 (69)	287 (96)	1.0	-	1.0	-
Yes	186 (31)	11 (4.0)	11.7 (6.2 to 22)	<0.001	3.7 (1.8 to 7.3)	<0.001
Missing	2 (0.3)	2 (0.7)	0.7 (0.1 to 4.9)	0.713	1.0	-
APGAR Score at 5 minutes						
≥9	352 (58)	290 (97)	1.0	-	1.0	-
<9	229 (38)	6 (2.0)	31.4 (14 to 72)	<0.001	15 (6.2 to 35)	<0.001
Missing	22 (3.7)	4 (1.3)	4.5 (1.5 to 13)	0.006	8.8 (1.1 to 70)	0.039

Data are N (%) or median (IQR) Abbreviations: CI, confidence interval; kg, kilogram; cm, centimetre; MUAC, mid-upper arm circumference; APGAR, appearance, pulse, grimace, activity, and respiration. ^a^Univariable logistic model for all cases vs. controls ^b^Multivariable logistic model for all cases vs. controls, including variables with P<0.1 ^c^Resuscitation using bag mask ventilation with oxygen and/or cardiopulmonary resuscitation

Among 502 EONS survivors and 101 EONS deaths, 141 (28%) and 38 (38%) respectively tested positive for at least one TAC target, whilst 30 (6.0%) and 7 (6.9%) tested positive for multiple targets. The most frequent organisms detected were *K. oxytoca*, *E. coli/Shigella* spp., *P. aeruginosa,* and *S. pyogenes* ( Table 3). Among 300 controls, 79 (26%) tested positive for at least one target, led by *K. oxytoca* and *P. aeruginosa*, and 11 (3.7%) were positive for multiple targets. *L. monocytogenes* and *M. tuberculosis* were not detected in either cases or controls. CMV was the commonest virus detected (19/603 (3.2%) cases versus 8/300 (2.7%) controls, p=0.7) and co-detection of CMV with a bacterial target was found in four cases and two controls.

**Table 3.** TaqMan results.

	Controls (n=300)	Cases (n=603)	Cases survived (n=502)	Cases died (n=101)	Cases vs Controls odds ratio (95% CI) ^a^	Cases (died) vs. Cases (survived) vs Controls cumulative odds ratio (95% CI) ^b^	Attributable Fraction among cases (95% CI) ^b^
**Bacteria**							
* Acinetobacter baumanii*	1 (0.3)	3 (0.5)	3 (0.6)	0 (0)	1.5 (0.2 to 14)	1.0 (0.3 to 3.6)	0
* Escherichia coli/Shigella spp.*	4 (1.3)	34 (5.6)	27 (5.4)	7 (6.9)	4.4 (1.6 to 13)	2.6 (1.6 to 4.4)	3.5 (1.7 to 5.3)
* Enterococcus faecalis*	0 (0)	4 (0.7)	3 (0.6)	1 (1.0)	-	4.0 (1.1 to 15)	0.5 (0.0 to 1.0)
* Haemophilus influenzae*	0 (0)	1 (0.2)	0 (0)	1 (1.0)	-	-	0.2 (0.0 to 0.5)
* Klebsiella oxytoca*	39 (13)	58 (9.6)	47 (9.4)	11 (11)	0.7 (0.5 to 1.1)	0.8 (0.5 to 1.2)	0
* Klebsiella pneumoniae*	1 (0.3)	9 (1.5)	7 (1.4)	2 (2.0)	4.5 (0.6 to 36)	2.7 (1.0 to 7.3)	0.9 (0.0 to 1.8)
* Listeria monocytogenes*	0 (0)	0 (0)	0 (0)	0 (0)	-	-	0
* Mycobacterium tuberculosis*	0 (0)	0 0)	0 (0)	0 (0)	-	-	0
* Neisseria meningitidis*	0 (0)	3 (0.5)	2 (0.4)	1 (1.0)	-	5.2 (1.0 to 28)	0.4 (0.0 to 0.9)
* Pseudomonas aeruginosa*	21 (7.0)	32 (5.3)	24 (4.8)	8 (7.9)	0.7 (0.4 to 1.3)	0.9 (0.5 to 1.6)	0
* Streptococcus agalactiae*	0 (0)	7 (1.2)	5 (1.0)	2 (2.0)	-	4.5 (1.6 to 13)	0.9 (0.2 to 1.6)
* Staphylococcus aureus*	1 (0.3)	1 (0.2)	1 (0.2)	0 (0)	0.5 (0.0 to 8.0)	0.4 (0.0 to 4.9)	0
* Streptococcus pneumoniae*	1 (0.3)	3 (0.5)	3 (0.6)	0 (0)	1.5 (0.2 to 14)	1.0 (0.3 to 3.6)	0
* Streptococcus pyogenes*	9 (3.0)	20 (3.3)	20 (4.0)	0 (0)	1.1 (0.5 to 2.5)	0.8 (0.5 to 1.4)	0
* Salmonella spp.*	1 (0.3)	3 (0.5)	2 (0.4)	1 (1.0)	1.5 (0.2 to 14)	2.0 (0.2 to 20)	0.2 (0.0 to 1.0)
* Ureaplasma spp.*	2 (0.7)	16 (2.7)	12 (2.4)	4 (4.0)	4.1 (0.9 to 18)	2.9 (1.3 to 6.4)	1.7 (0.5 to 3.0)
Any bacteria	72 (24)	156 (26)	123 (25)	33 (33)	1.1 (0.8 to 1.5)	1.2 (0.9 to 1.6)	4.5 (0.0 to 11)
Any bacteria excluding *K. oxytoca* and *P. aeruginosa*	20 (6.7)	89 (15)	70 (14)	19 (19)	2.4 (1.5 to 4.0)	2.1 (1.5 to 3.1)	7.9 (4.3 to 11)
**Viruses**							
Cytomegalovirus	8 (2.7)	19 (3.2)	15 (3.0)	4 (4.0)	1.2 (0.5 to 2.7)	1.3 (0.6 to 2.7)	0.6 (0.0 to 2.7)
Enterovirus	0 (0)	6 (1.0)	3 (0.6)	3 (3.0)	-	9.1 (2.3 to 37)	0.9 (0.2 to 1.6)
Herpes Simplex Virus 1	1 (0.3)	1 (0.2)	1 (0.2)	0 (0)	0.5 (0.0 to 8.0)	0.4 (0.0 to 4.9)	0
Herpes Simplex Virus 2	1 (0.3)	1 (0.2)	1 (0.2)	0 (0)	0.5 (0.0 to 8.0)	0.4 (0.0 to 4.9)	0
Any viruses	10 3.3)	27 (4.5)	20 (4.0)	7 (6.9)	1.4 (0.6 to 2.8)	1.6 (0.8 to 3.2)	1.6 (0.0 to 3.9)
**Protozoa**							
* Plasmodium* spp.	0 (0)	7 (1.2)	6 (1.2)	1 (1.0)	-	2.9 (1.4 to 6.2)	0.8 (0.1 to 1.4)
**Any bacteria, viruses and** **protozoa**	79 (26)	179 (30)	141 (28)	38 (38)	1.2 (0.9 to 1.6)	1.3 (1.0 to 1.7)	6.6 (0.0 to 14)
**Any bacteria (excluding** ***K. oxytoca* and *P. aeruginosa*),** **viruses and protozoa**	30 (10)	115 (19)	91 (18)	24 (24)	2.1 (1.4 to 3.3)	2.0 (1.4 to 2.8)	9.4 (5.1 to 13)

^a^Ordinary logistic regression ^b^Ordinal logistic regression ^c^Attributable fraction is calculated from cumulative odds ratio

**Figure 3.**
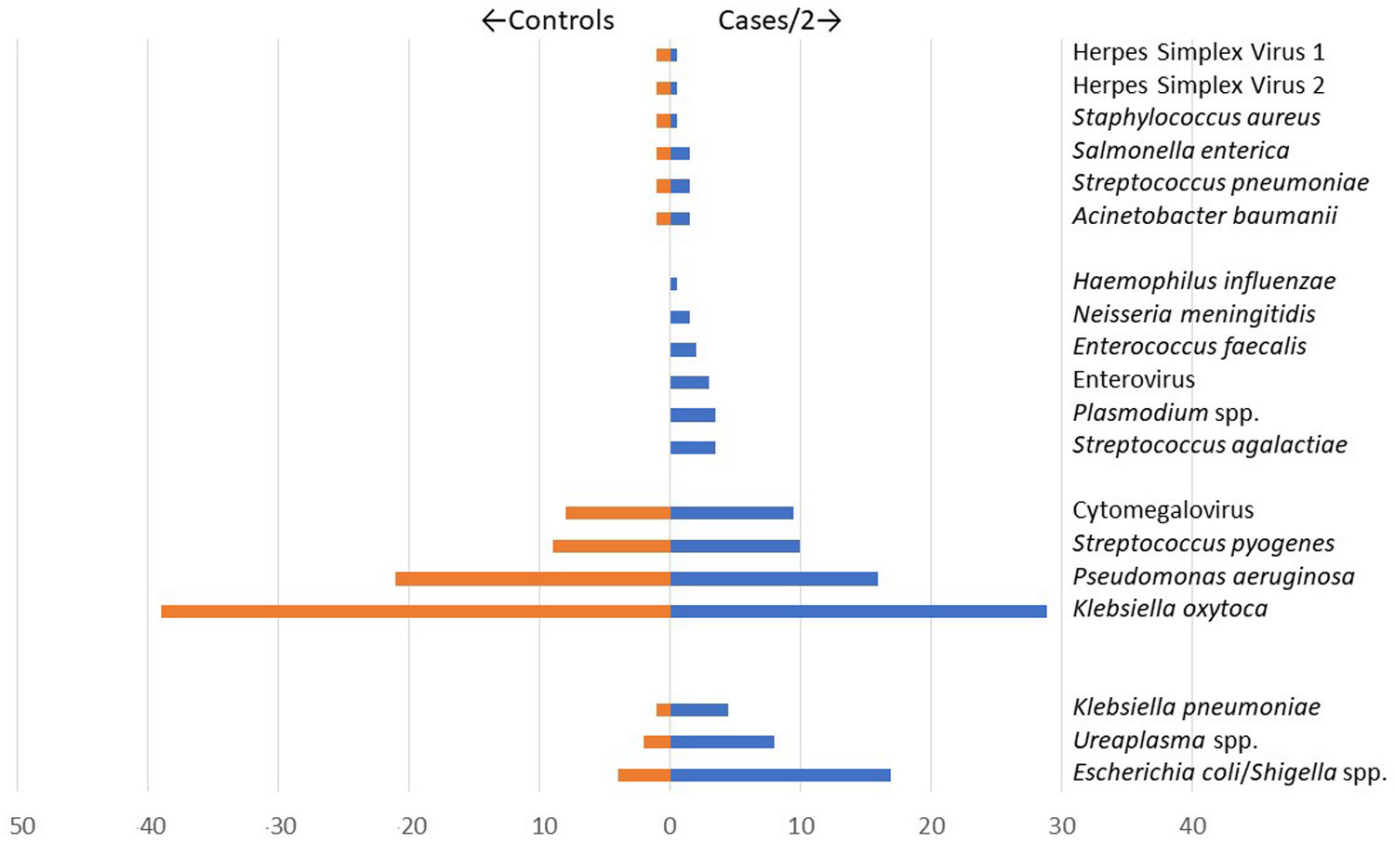
Patterns of detection of Taqmann PCR Targets. Organisms included in the TAC were detected in 4 distinct groups: Group 1 (Herpes Simplex Virus 1, Herpes Simplex Virus 2, *Staphylococcus aureus*, *Salmonella enterica*, *Streptococcus pneumoniae*, and *Acinetobacter baumanii*); Group 2 ( *Hemophilus influenzae*, *Neisseria meningitidis*, *Enterococcus faecalis*, Enterovirus, *Plasmodium* spp., and *Streptococcus agalactiae*); Group 3 (Cytomegalovirus, *Streptococcus pyogenes*, *Pseudomonas aeruginosa*, and *Klebsiella oxytoca*); and Group 4 ( *Klebsiella pneumoniae, Ureaplasma* spp. and *Escherichia coli/Shigella* spp.). Weighting of cases (represented as cases/2) was done to allow for improved accuracy in assessing the distribution of organisms tested, comparing cases to controls, since cases (n=603) were ~twice more than controls (n=300). Weighting was done by calculating the number of eligible cases/controls divided by the number of enrolled cases/controls i.e. the inverse of the sampling fraction for cases/controls.

We observed four patterns of target detection by TAC ( Figure 3):

i)    Group 1: detected in a low proportion (<5.0%) in both cases (12/603 [2.0%]) and controls (6/300 [2.0%]), ((HSV 1, HSV 2, *S. aureus*, *Salmonella enterica*, *S. pneumoniae*, and *A. baumanii*);

ii)    Group 2: detected in a low proportion (<5.0%) in cases (27/603 [4.5%]) but none in controls (0/300 [0.0%]), ( *H. influenzae*, *N. meningitidis*, *E. faecalis*, Enterovirus, *Plasmodium* spp., and *S. agalactiae*);

iii)    Group 3: detected in a high proportion (≥5.0%) in both cases (123/603 [20%]) and controls (70/300 [23%]), (CMV, *S. pyogenes*, *P. aeruginosa*, and *K. oxytoca*); and

iv)    Group 4: detected in a high proportion (≥5%) in cases (47/603 [7.8%]) and low proportion (<5.0%) in controls (6/300 [2.0%]) ( *K. pneumoniae*, *Ureaplasma* spp. and *E. coli/Shigella* spp.).

Upon examining cumulative odds, detection of any bacterial, viral or protozoal target was not associated with pSBI and death (OR 1.3, 95% CI 1.0-1.7) ( Table 3). However, the high proportions of *K. oxytoca* and *P. aeruginosa* in both cases and controls suggested contamination or clinically insignificant traces of DNA in blood. Excluding these organisms, detection of any target was associated with pSBI and death (OR 2.1, 95% CI 1.4-2.8).

*E. coli/Shigella* spp. (P *<*0.001), *E. faecalis* (P *=*0.034), *S. agalactiae* (P *=*0.004), *Ureaplasma* spp. (P *=*0.010), Enterovirus (P *=*0.002), and *Plasmodium* spp. (P *=*0.004) were associated with pSBI and death ( Table 3). *K. pneumoniae* (P *=*0.050) and *N. meningitidis* (P *=*0.054) had P values of borderline significance.

Overall, 6.6% (95% CI 0-14) of all pSBI cases were attributed to the bacterial, viral or protozoal targets. Excluding *K. oxytoca* and *P. aeruginosa* as likely contaminants, 9.4% (95% CI 5.1-13) of cases were attributed to the tested targets. Overall, 4.5% (95% CI 0-11) and 1.6% (95% CI 0-3.9) of pSBI cases were attributed to bacterial and viral targets respectively. The leading attributed pathogens were *E. coli/Shigella* spp. (AF 3.5%, 95% CI 1.7-5.3) and *Ureaplasma* spp. (AF 1.7, 95% CI 0.5-3.0). *E. faecalis*, *H. influenzae*, *K. pneumoniae*, *N. meningitidis*, *S. agalactiae*, *Salmonella enterica*, CMV, Enterovirus and *Plasmodium* spp. were each attributed to less than 1% of the pSBI cases.

A total of 11 (1.8%) of 603 cases had presumed pathogens isolated from blood culture at admission and *S. aureus* (n=5) was the most common isolate ( Table 4). The OR for a positive admission blood culture for death among admitted pSBI cases was 1.1 (95% CI 0.24-5.2) and 0.2% (95% CI 0-3.2) of pSBI were attributed to the pathogens identified through blood culture.

**Table 4.** Admission blood culture results among cases and corresponding TAC PCR results.

	Cases (n=603)	
Organisms	Blood culture	TAC cord blood
Presumed significant organisms		
*Acinetobacter* spp.	2	0
*Klebsiella pneumoniae*	1	0
*Pantoea* spp. ^*^	1	0
*Staphylococcus aureus*	5	1 ( *K. oxytoca* + *P. aeruginosa*)
*Streptococcus* Group B	1	1 (CMV)
*Streptococcus* Group G ^*^	1	1 ( *K. oxytoca*)
Total	11	3

Columns show number of neonates with either positive culture or TAC (TAC organisms are indicated within the brackets). Abbreviations: CMV, cytomegalovirus. *These organisms were not included on the TAC.

## Discussion

We used a novel approach to identify causes of EONS by investigating stored cord blood samples collected at birth with a custom TAC, spatially multiplexed PCR to interrogate the presence of multiple pathogens. This is the first study evaluating diagnostic performance using cord blood in an African setting. These samples were obtained at delivery prior to admission with signs of pSBI. Approximately 60% of 603 EONS cases and 92% of all deaths were admitted on day 0 of life. A total of 58 of 101 (57%) deaths occurred within the first 24 hours of life, after cord blood samples had been obtained. This underscores the importance of prompt diagnosis for targeted treatment and makes a cord blood approach potentially attractive in epidemiological studies, and possibly for managing ‘at risk’ neonates since cord blood could be collected, stored and tested at a later stage if the newborn develops signs of pSBI.

*E. coli/Shigella* spp. and *Ureaplasma* spp. had the highest causal attribution in our results, supporting the latter as an important pathogen in this setting. *Ureaplasma* spp. are associated with maternal colonization and adverse pregnancy outcomes ^46^. Appropriate treatment of high-risk neonates is needed given the emerging resistance of *Ureaplasma* spp. to macrolides. Two healthy controls and 16 pSBI cases, of whom 12 survived, tested positive for *Ureaplasma* spp. in our study. Although the clear association of *Ureaplasma* spp. with sepsis and mortality indicates pathogenicity, asymptomatic presentation or recovery in ill neonates without targeted antimicrobials has been reported and is not unusual ^47^. Additionally, the culture-independent molecular method identified non-culturable organisms such as Enterovirus, which is shown to cause serious sepsis-like illness in neonates in other settings ^48^. However, despite the use of sensitive molecular assays, 90% of pSBI cases still had unknown aetiology on cord blood analysis.

The overall causal attribution of 6.6% (95% CI 0-14) increased to 9.4% (95% CI 5.1-13) with exclusion of *K. oxytoca* and *P. aeruginosa* in our study. As expected, the attributable proportion was lower than in the Sepsis Aetiology in Neonates in South Africa study (SANISA [27%, 95% CI 23-32]) ^27^ and the Aetiology of Neonatal Infection in South Asia study (ANISA [23%, 95% CI 19-26]) ^28^ since much of the latter study’s attribution went to detection of RSV in respiratory samples, which this study did not examine. Although SANISA (n=27) and ANISA (n=28) were large prospective studies and tested more targets by TAC than we did (n=21), they also failed to attribute aetiology to a large proportion of pSBI cases. There were also differences in pSBI case definitions (SANISA used a predefined set of clinical and laboratory criteria ^27^, while ANISA used WHO clinical criteria but excluded tachypnoea ^28^) and differences in selection and sampling of controls (SANISA sampled healthy neonates at study hospital ^27^ while ANISA used an automated algorithm triggered at the first postnatal visit to select randomly registered controls ^28^). Our pSBI definition was based on the WHO Young Infants Clinical Signs study which derived a decision rule (presence of ≥1 sign: history of difficulty feeding, history of convulsions, movement only when stimulated, respiratory rate of ≥60 breaths/min, severe chest indrawing, and a temperature of ≥37.5°C, or ≤35.5°C) predicting severe illness in neonates aged 0-6 days with 87% sensitivity and 74% specificity ^39^. The performance of these signs in distinguishing neonates with sepsis from those without sepsis has not been adequately investigated. Current WHO ^49^ and Kenya national paediatric guidelines ^50^ for empiric antimicrobials in neonates suspected to have sepsis are based on this limited evidence. Neonates with sepsis often present with subtle and non-specific clinical signs that overlap with those seen in other non-infectious diagnoses ^51^. Thus, our case definition may have resulted in the inclusion of neonates who did not have true sepsis, contributing to low attribution rates. Development and use of a highly sensitive and specific consensus definition for neonatal sepsis is critically needed in clinical practice and research ^6^.

Bacterial organisms (25% bacterial compared to 4.1% viral targets detected) were predominant in our study, similar to SANISA ^27^ and ANISA ^28^ results from blood samples. Thirty percent of cord blood samples of pSBI cases in our study tested positive for at least one target by TAC, compared to blood samples in SANISA (37%) ^27^ and ANISA (12%) ^28^. We identified multiple targets in 7% of pSBI cases compared to 11% cases in SANISA ^27^ and 1% in ANISA ^28^. At least one target was positive in 28% of healthy controls in our study compared to 20% in SANISA ^27^. Thus, background positivity of cord blood among healthy neonates in our study was greater than in SANISA. All cases and controls were first selected based on the presence or absence of pSBI. All 604 pSBI cases who resided in the KHDSS had cord blood samples available for testing and were included in this analysis. 300 controls were randomly selected from a subset of 6,295 neonates who were resident of the KHDSS, remained well during the first 60 days of life, and had cord blood samples available for testing. Therefore, we ensured that cases and controls had an equal chance of being selected in respective groups, with controls derived from similar circumstances to cases for optimal group comparison, unlike in ANISA where controls were recruited from the community. Although the cases had a lower gestation age and were generally smaller than the controls based on the anthropometric measurements, we believe that bias risk was minimal since we would expect sick neonates to present with known underlying risk factors of infection, such as prematurity.

*K. oxytoca* and *P. aeruginosa* were identified in large numbers in both cases and controls. This could be due to environmental contamination of laboratory materials or reagents, which has been widely reported for *K. oxytoca* ^52^, contamination of the specimen by gut flora or skin commensals post-delivery ^53^, as was reported in SANISA ^27^, or true subclinical detection of circulating non-viable genetic material, or low copies of organisms insufficient to cause disease. Overall causal attribution increased with exclusion of *K. oxytoca* and *P. aeruginosa*, suggesting non-significance of these bacteria in EONS. Cord blood sample contamination has been reported in studies evaluating the diagnostic use of cord blood cultures, by comparing results obtained to peripheral venous blood cultures ^29, 33, 35^. Although cord blood provides a non-invasive alternative to peripheral blood sampling with better culture yields ^30, 54^, the risk of contamination cannot be ignored. Careful aseptic techniques and training of clinical staff are imperative to optimize sample collection and may improve the validity of results. Aseptic techniques were used during cord blood sample collection to minimise sample contamination, since identification of pathogens associated with adverse maternal and perinatal outcomes was planned ^36^, including a recently published study on the association of flavivirus exposure with congenital microcephaly ^55^. In addition, cord blood analysis using PCR has mostly focused on vertically transmitted viruses ^56– 58^, and more research on cord blood testing using molecular diagnostics is needed to better understand the clinical significance of detected organisms. Detection of organisms known to cause permanent neurodevelopmental sequelae in asymptomatic congenital infection such as CMV ^59^ (eight healthy controls in our study) may inform management. However, we did not follow up these infants for post-discharge outcomes in this retrospective analysis. Nonetheless, the PCR detections for *E. coli/Shigella* spp., *E. faecalis*, *K. pneumoniae*, *N. meningitidis*, *S. agalactiae*, *Ureaplasma* spp., and *Plasmodium* spp. had clear directional association across controls, surviving cases, and cases who died.

A limitation of this study was that we could not rigorously compare cord blood PCR to cord blood culture since we did not have paired specimens. Blood culture was performed on later specimens at ward admission. Although blood culture is the gold standard test for sepsis, culture-negative neonatal sepsis is common ^19^, and this is evident in the low positivity rate among the pSBI cases in our study. In addition, the tests differed in the volumes of blood used for processing (0.1 ml equivalent per PCR reaction versus ~2ml for culture ^60^) and timing of testing (immediately for culture, stored for ~5 years for TAC). Low burden of infection at the limit of detection, different sampling timepoints, and decreased *S. agalactiae* sampling sensitivity due to antisepsis measures associated with caesarean section delivery ^14^, may have contributed to failure to detect *S. agalactiae* by cord blood TAC, in a pSBI case from whom *S. agalactiae* was isolated from admission blood culture five hours after delivery.

Maternal variables at delivery can aid prompt initiation of antimicrobials. Intrapartum fever (temperature ≥38°C), chorioamnionitis, pre-labour rupture of membranes ≥18 hours, preterm pre-labour rupture of membranes, PROM ≥18 hours, maternal GBS colonization or bacteriuria, multiparity, and poor intrapartum and postpartum infection control practices have previously been shown to predispose neonates to infection ^5, 61, 62^. We lacked complete data on intrapartum antibiotic use and were unable to assess its impact on pathogen identification. In addition to an immunological immaturity ^63^, prematurity, low birth weight, complicated or instrument-assisted delivery, and low APGAR scores, contribute to an increased risk of admission with EONS. Although not the primary aim of our study, we observed that being identified as very preterm (<32 weeks) as well as head circumference and MUAC, which are associated with maturity ^64^, were associated with EONS. However, low birth weight was not associated with EONS in our study.

Although TAC provided epidemiological data on potential causes of EONS in our setting, including the role of nonculturable organisms such as *Ureaplasma* spp. and Enterovirus, 90% of pSBI cases lacked epidemiological attribution. The presence of presumed contaminants in both cases and controls was only discernible on a population basis rather than from an individual’s results. Thus, despite allowing for customization of a panel of pathogen targets, requirement of small blood volumes, and rapid pathogen detection, TAC in its current form may have a limited role in individual diagnosis in clinical practice, particularly in settings like ours where associated costs of setting up and using this platform will be prohibitive. Further research using this technology alongside highly specific diagnostic methods is needed to better understand the aetiology, distribution and determinants of disease. In addition, our study was limited by use of archived samples and retrospective analysis of data. Future prospective studies using specific definitions of EONS alongside paired cord blood and peripheral blood cultures are needed to better understand the performance of TAC in detection of pathogens associated with EONS.

In conclusion, we were able to identify organisms associated with subsequent EONS and death using cord blood at birth and an identically sampled comparator group of healthy neonates in sub-Saharan Africa. Further prospective research on the clinical utility of cord blood in our setting is needed alongside development and use of rapid and specific point-of-care diagnostics, that will guide prompt management in seriously ill neonates. Robust evidence of the causes of EONS is vital, given the potential for prevention and targeted treatment strategies such as maternal immunization and intrapartum antibiotic prophylaxis ^65^, including oral azithromycin for reduction of bacterial carriage and risk of EONS ^66^. Coverage for *Ureaplasma* spp. in at-risk neonates should be considered when updating antimicrobial guidelines given the strength of combined data from three studies (ours, SANISA and ANISA) and the potential adverse outcomes associated with this organism ^67^. 

## Data availability

### Underlying data

Harvard Dataverse: Replication Data for: Detection of pathogens associated with early-onset neonatal sepsis in cord blood at birth using quantitative PCR, https://doi.org/10.7910/DVN/FXKGRB ^68^

This project contains the following underlying data:

- Maternal variables-1.tab
- Neonatal variables-1.tab
- PCR Ct values-1.tab

### Extended data

Harvard Dataverse: Replication Data for: Detection of pathogens associated with early-onset neonatal sepsis in cord blood at birth using quantitative PCR, https://doi.org/10.7910/DVN/FXKGRB ^68^

This project contains the following extended data:

- CObiero_Detection of pathogens in cord blood_Codebook.pdf
- CObiero_Detection of pathogens in cord blood_readme.txt
- Detection of pathogens at birth_Extended data.pdf

Data are available under the terms of the Creative Commons Attribution 4.0 International license (CC-BY 4.0).
