# Supplementary material for: Detection of pathogens associated with early-onset neonatal sepsis in cord blood at birth using quantitative PCR
Source: Wellcome Open Res. 2022 Nov 8;7:3. Originally published 2022 Jan 5. [Version 3] doi: 10.12688/wellcomeopenres.17386.3 (PMC9114825; doi:10.12688/wellcomeopenres.17386.3)
Supplement: Peer review history

# Peer review: Reviewer response for version 3

**DOI:** 10.21956/wellcomeopenres.19881.r53199

**Published:** 2022-11-09

**Authors:** Kirsty Le Doare

**Affiliations:** 1 Paediatric Infectious Diseases Research Group and Vaccine Institute, Institute of Infection and Immunity, St George's University of London, London, UK

All changes look appropriate. I maintain my status of 'Approved'.

Is the work clearly and accurately presented and does it cite the current literature?

Yes

If applicable, is the statistical analysis and its interpretation appropriate?

Yes

Are all the source data underlying the results available to ensure full reproducibility?

Yes

Is the study design appropriate and is the work technically sound?

Yes

Are the conclusions drawn adequately supported by the results?

Yes

Are sufficient details of methods and analysis provided to allow replication by others?

Yes

Reviewer Expertise:

Neonatal infection, vaccines, antimicrobial resistance, global health

I confirm that I have read this submission and believe that I have an appropriate level of expertise to confirm that it is of an acceptable scientific standard.

# Peer review: Reviewer response for version 2

**DOI:** 10.21956/wellcomeopenres.19812.r50517

**Published:** 2022-05-17

**Authors:** Kirsty Le Doare

**Affiliations:** 1 Paediatric Infectious Diseases Research Group and Vaccine Institute, Institute of Infection and Immunity, St George's University of London, London, UK

This study from Kenya investigated early-onset sepsis over a 5 year period using the TaqMan array comparing cases and controls. Such studies are much needed as there is very little information about early-onset aetiology in African countries.

 The manuscript is well written and there are only a few minor points to consider:

1. The global health community is moving away from using terms such as “sub-Saharan Africa” and “developing countries” in preference for “African countries” and “resource-limited settings”, as there is a lot of heterogeneity between countries in SSA in terms of development and resources.
2. Methods – Were antibiotics, in labour for example, because of caesarian section monitored? As this could account for the fairly low positivity of results, even by PCR.
3. Methods – How were cases and controls selected? Was any sensitivity analysis undertaken to look for potential bias in case selection?
4. Results – How did the authors account for the fact that the majority of pathogens were not identified by culture?
5. Results – Is it possible to look at the *S. epi *as a potential pathogen if any of the infants were premature of VLBW?
6. Discussion – As with all such studies, relating DNA/RNA to clinical presentation is difficult and the authors have found >1 pathogen in many cases, so it is still difficult to identify causative agents. On the other hand, the fact that the same pathogens appear as have been found in culture but that more fastidious bacteria (ureaplasma) is found on PCR highlights both the need for a better method of assessing sepsis in infants and the need for more prospective studies using molecular methods to identify causation.

Is the work clearly and accurately presented and does it cite the current literature?

Yes

If applicable, is the statistical analysis and its interpretation appropriate?

Yes

Are all the source data underlying the results available to ensure full reproducibility?

Yes

Is the study design appropriate and is the work technically sound?

Yes

Are the conclusions drawn adequately supported by the results?

Yes

Are sufficient details of methods and analysis provided to allow replication by others?

Yes

Reviewer Expertise:

Neonatal infection, vaccines, antimicrobial resistance, global health

I confirm that I have read this submission and believe that I have an appropriate level of expertise to confirm that it is of an acceptable scientific standard.

# Response

**Published:** 2022-05-27

**Authors:** Christina Obiero

**Affiliations:** Kenya Medical Research Institute, Kenya

This study from Kenya investigated early-onset sepsis over a 5-year period using the TaqMan array comparing cases and controls. Such studies are much needed as there is very little information about early-onset aetiology in African countries.

 The manuscript is well written and there are only a few minor points to consider:

 1. The global health community is moving away from using terms such as “sub-Saharan Africa” and “developing countries” in preference for “African countries” and “resource-limited settings”, as there is a lot of heterogeneity between countries in SSA in terms of development and resources.

*Response: Thank you for this important comment. We acknowledge that countries in Africa have different levels of social, economic, environmental, and political development and this plays a role in health service delivery and outcomes. In addition, populations residing in African countries face regional health disparities due to unequitable distribution of resources. In our manuscript, we used the term “sub-Saharan Africa” to refer to data on the aetiology and burden of neonatal sepsis obtained from studies conducted in this region that is distinct from African countries within the Sahara, and other resource-limited settings in Asia and the Middle East.*

 2. **Methods** – Were antibiotics, in labour for example, because of caesarian section monitored? As this could account for the fairly low positivity of results, even by PCR.

*Response: Mothers presenting with signs of infection during labour at Kilifi County Hospital (KCH) are treated with antibiotics. Screening for Group B Streptococcus and peripartum antibiotic prophylaxis are not done at KCH. During the Kilifi Perinatal and Maternal Surveillance (KIPMAT), data on antibiotics used within 4 weeks of labour was collected on a standardised maternal admission record as described in the manuscript. If a mother received antibiotics, clinicians documented whether this was indicated for premature rapture of membranes or not. Data on antibiotic use was not complete as indicated in the discussion section and we were, therefore, unable to assess the impact of antibiotic pretreatment on PCR and culture (cases only) results.*

*Discussion, page 22: “ **We lacked complete data on intrapartum antibiotic use and were unable to assess its impact on pathogen identification.**”*

 3. **Methods** – How were cases and controls selected? Was any sensitivity analysis undertaken to look for potential bias in case selection?

*Response: Cases and controls were selected from a cohort of 15,409 neonates born at KCH during the study period. Cases included in the analysis were residents of the Kilifi Health and Demographic Surveillance System (KHDSS) and were hospitalised at KCH within the first 48 hours of life with signs of World Health Organisation-defined possible serious bacterial infection (pSBI). Controls were also KHDSS residents, discharged home well after delivery, and not hospitalised/remained alive during the first 60 days of life. Both cases and controls needed to have adequate volumes of cord blood samples available for analysis. All 604 cases whose cord blood samples were tested had cord blood samples available. In addition to meeting the criteria for being a control, including having cord blood samples available for analysis, the 300 controls in our study were randomly selected from a cohort of 6,295 healthy neonates. Selection bias cannot be completely ruled out especially since those with available cord blood samples were included while some neonates with available cord blood samples were excluded from our analysis. However, we were unable to perform a sensitivity analysis, e.g. testing of samples obtained from neonates who had available samples but were not included in the analysis as this would have required additional diagnostic resources. We have added this as a limitation as shown below:*

*Discussion, page 22: “* ***Sensitivity analysis of neonates excluded in our study despite having available cord blood samples, some of which were of low volumes would have been useful in checking for any selection bias in our case-control selection, and in comparing our PCR results.*** *”*

 4. **Results** – How did the authors account for the fact that the majority of pathogens were not identified by culture?

*Response: Thank you for this important comment. Only 1.8% of blood cultures done among 603 pSBI cases were positive for known pathogens. None of the controls had cultures done as they were discharged home well soon after birth. Our low culture yield is consistent with that in similar settings. This is because of factors such as poor culture sensitivity, low sample volumes (secondary to difficult venepuncture among neonates), and (maternal) antibiotic pre-treatment. In addition, some of the pathogens detected by TAC are fastidious/unculturable and hence were not identified by culture. Discordance between TAC PCR and culture results was reported in the ANISA and SANISA studies. In our study, TAC PCR and culture samples were collected and tested under different circumstances, as described in the discussion. In addition, we proposed that future studies testing paired cord blood and peripheral venous blood samples using advanced molecular tests and comparing these to culture will be more informative on the aetiology of sepsis and the performance of these tests.*

*Discussion, page 22: “A limitation of this study was that we could not rigorously compare cord blood PCR to cord blood culture since we did not have paired specimens. **However, studies such as ANISA and SANISA which performed blood TAC PCR and culture in parallel reported discordance of results between the two tests. In our study,** **b**lood culture was performed on later specimens at ward admission. Although blood culture is the gold standard test for sepsis, culture-negative neonatal sepsis is common ^19^, and this is evident in the low positivity rate among the pSBI cases in our study. In addition, the tests differed in the volumes of blood used for processing (0.1 ml equivalent per PCR reaction versus ~2ml for culture ^61^) and timing of testing (immediately for culture, stored for ~5 years for TAC). Low burden of infection at the limit of detection, different sampling timepoints, and decreased S. agalactiae sampling sensitivity due to antisepsis measures associated with caesarean section delivery ^14^,may have contributed to failure to detect S. agalactiae by cord blood TAC, in a pSBI case from whom S. agalactiae was isolated from admission blood culture five hours after delivery. **In addition, some of the pathogens were fastidious/unculturable and were only detected by TAC.** Low aetiological attribution by culture among pSBI cases underscores the need for better diagnostics as bacteraemia was associated with an increased likelihood of case fatality.”*

 5. **Results** – Is it possible to look at the S. epi as a potential pathogen if any of the infants were premature of VLBW?

*Response: While customising the TaqMan Array Card (TAC), we reviewed the literature and considered organisms shown to cause neonatal sepsis in previous studies as indicated on page 7 (Detection of targets using TAC RT-qPCR). S. epidermidis is a common culture contaminant in different settings including ours. It was the leading admission blood culture contaminant (n=27) among all pSBI cases. We previously examined systematic clinical and microbiologic surveillance data from all neonatal admissions (including premature neonates) to KCH to determine the association of Coagulase-negative Staphylococci (CoNS [including S. epidermidis]) with case fatality and/or prolonged duration of admission among neonates.* *^1^* *We found that CoNS had no clinical significance in our setting where long intravascular lines and invasive ventilation are not available, hence not requiring targeted antibiotic treatment. We revised the section describing the selection of TAC targets as follows:*

***"Detection of targets using TAC RT-qPCR, page 7: “Organisms such as CoNS that have been previously shown to be clinically insignificant in our setting ^44^ were not included in the TAC panel.”***

 6. **Discussion** – As with all such studies, relating DNA/RNA to clinical presentation is difficult and the authors have found >1 pathogen in many cases, so it is still difficult to identify causative agents. On the other hand, the fact that the same pathogens appear as have been found in culture but that more fastidious bacteria (ureaplasma) is found on PCR highlights both the need for a better method of assessing sepsis in infants and the need for more prospective studies using molecular methods to identify causation.

*Response: Thank you for this important observation. Aetiological attribution of pathogens to infection is challenging, especially where multiple organisms are detected. This has been shown in several studies (e.g. ANISA and SANISA) investigating the aetiology of infection and using advanced statistical analysis, e.g. Bayesian latent class models. This demonstrates the need for more advanced diagnostics, such as the use of pathogen-specific biomarkers and metagenomics, to better understand the epidemiology of neonatal sepsis, given the large proportion of cases lacking causal attribution following the use of TAC reverse-transcription quantitative polymerase chain reaction.*

**Reference**

 1. Seale AC, Obiero CW, Jones KD, et al. Should First-line Empiric Treatment Strategies for Neonates Cover Coagulase-negative Staphylococcal Infections in Kenya? *The Pediatric infectious disease journal* 2017;36(11):1073-78. doi: 10.1097/INF.0000000000001699 [published Online First: 2017/07/22]

# Peer review: Reviewer response for version 1

**DOI:** 10.21956/wellcomeopenres.19223.r49589

**Published:** 2022-04-19

**Authors:** Kari A. Neemann

**Affiliations:** 1 Pediatrics, Division of Infectious diseases, Children's Hospital and Medical Center, University of Nebraska Medical Center, Omaha, NE, USA

I would like to commend the authors for preparing a well-cited and thoughtful interpretation of their results. Overall, it is hard what to make of this data in the clinical setting.  None of the potential identified pathogens on the cord blood sample were identified by the traditional “gold standard” blood culture which even with its limitations is hard to account for. A large percentage of neonates in both the cases and controls had ≥1 typical pathogen identified ( *K. oxytoca, Streptococcus pyogenes, *and *P. aeruginosa*); organisms which, with the noted exception for *K oxytoca*, are not typically considered contaminants. I agree with the authors that further prospective studies utilizing paired samples of cord blood multiplex PCR, cord blood culture, and peripheral blood cultures in EONS are needed to fully evaluate the utility of this assay in the clinical setting.

 Materials and Methods:

1. “Cases were defined as neonates hospitalized within 48 hours of life with one or more features of pSBI.” Please confirm that none of the cases were discharged from the hospital and re-admitted within 48 hours of life.
2. While Staphylococcus epidermidis is a common contaminant in most populations, it can represent a true pathogen in neonates though admittedly more so as late-onset sepsis in VLBW infants. Was there any thought to including this as a pathogen?
3. Did every neonate with pSBI have a blood culture obtained? While it is mentioned as routine clinical care it is not clear whether this occurred or not. Try to determine how to interpret the positive blood culture results in the results sections. 

Results: 

1. 'Table 1. Prolonged rapture of membranes' - change *rapture *to *rupture*. 
2. Figure 3. Would label on the right Groups 1-4.

Discussion:

1. I thought the authors did a good job of describing the strengths and limitations of the study and putting this study into the context of the ANISA and SANISA trials. 

Is the work clearly and accurately presented and does it cite the current literature?

Yes

If applicable, is the statistical analysis and its interpretation appropriate?

Yes

Are all the source data underlying the results available to ensure full reproducibility?

Yes

Is the study design appropriate and is the work technically sound?

Yes

Are the conclusions drawn adequately supported by the results?

Yes

Are sufficient details of methods and analysis provided to allow replication by others?

Yes

Reviewer Expertise:

Pediatric Infectious Diseases

I confirm that I have read this submission and believe that I have an appropriate level of expertise to confirm that it is of an acceptable scientific standard.

# Response

**Published:** 2022-05-03

**Authors:** Christina Obiero

**Affiliations:** Kenya Medical Research Institute, Kenya

*Many thanks for the comments on this manuscript. We have revised the manuscript based on reviewer’s comments and responded to specific points as below.*

**Materials and Methods:**

 1. “Cases were defined as neonates hospitalized within 48 hours of life with one or more features of pSBI.” Please confirm that none of the cases were discharged from the hospital and re-admitted within 48 hours of life.

*Response: Thank you for this important comment. All cases included in this analysis were admitted within the first 48 hours of life. None of the pSBI cases were discharged home from the hospital and readmitted within the first 48 hours of life. Unique identifiers are used at our hospital for each admission and laboratory sample, and none of the cases had more than one episode of admission during the first 60 days of life, including the first 48 hours of life when they developed signs of pSBI and were hospitalised. We have clarified this in the relevant section as shown below:*

*Study design and participants, Page 6: **“None of the cases were readmitted following the initial hospital admission, including during the first 48 hours of life.”***

 2. While *Staphylococcus epidermidis* is a common contaminant in most populations, it can represent a true pathogen in neonates though admittedly more so as late-onset sepsis in VLBW infants. Was there any thought to including this as a pathogen?

*Response: While customising the TaqMan Array Card (TAC), we reviewed literature and considered organisms shown to cause neonatal sepsis in previous studies as indicated on page 8 (Detection of targets using TAC RT-qPCR). S. epidermidis is a common culture contaminant in different settings including ours. It was the leading admission blood culture contaminant (n=27) among all pSBI cases. We previously examined systematic clinical and microbiologic surveillance data from all neonatal admissions to Kilifi County Hospital to determine association of Coagulase-negative Staphylococci (CoNS [including S. epidermidis]) with case fatality and/or prolonged duration of admission among neonates and found that CoNS were not clinically significant organisms in our setting where long intravascular lines and invasive ventilation are not available, hence not requiring targeted antibiotic treatment.* *^1^* *In addition, our clinical setting lacks factors that often predispose patients to colonization and invasion by CoNS e.g. use of indwelling medical devices. We have revised the section describing the selection of TAC targets as follows:*

*Detection of targets using TAC RT-qPCR, page 8: **“Organisms such as CoNS that have been previously shown to be clinically insignificant in our setting ^44^ were not included in the TAC panel.”***

 3. Did every neonate with pSBI have a blood culture obtained? While it is mentioned as routine clinical care it is not clear whether this occurred or not. Try to determine how to interpret the positive blood culture results in the results sections.

*Response: Thank you for this important comment. All neonates with pSBI had blood culture done at admission for clinical care and as part of ongoing clinical surveillance for bacteraemia on the ward. We have clarified this section as follows:*

*Study design and participants, page 6: “Routine laboratory investigations **for all admissions** for clinical care included blood culture (BACTEC Peds Plus/F bottles and BACTEC 9050 instrument, Becton Dickinson, UK) and cerebrospinal fluid (CSF) culture where indicated, as previously described ^41^.”*

*All 603 pSBI cases included in this analysis had blood cultures done at admission. Eleven (1.8%) neonates had presumed pathogens (led by Staphylococcus aureus [n=5]) isolated from blood culture while 37 (6.1%) had contaminants (led by S. epidermidis [n=27]).* *Cord blood samples and admission blood culture samples were obtained at different timepoints and correlation of these two results was not possible in our study as discussed on page 15. In the results section, we indicated that* t *he odds ratio for a positive admission blood culture for death among admitted pSBI cases was 1.1 (95% CI 0.24-5.2) and 0.2% (95% CI 0-3.2) of pSBI were attributed to the pathogens identified through blood culture. This means that although bacteraemia was associated with increased mortality among pSBI cases, culture yields were low and provided little information about pathogens causing morbidity and death among these hospitalised neonates. We have updated the discussion section as follows:*

*Discussion, page 15: **“Low aetiological attribution by culture among pSBI cases underscores the need for better diagnostics as bacteraemia was associated with an increased likelihood of case fatality.”***

**Results:**

 1. 'Table 1. Prolonged rapture of membranes' - change rapture to rupture.

*Response: Thank you for this helpful observation. We have corrected the spelling as required.*

 2. Figure 3. Would label on the right Groups 1-4.

*Response: Thank you for this helpful suggestion. We have labelled the groups on Figure 3 appropriately.*

**Discussion:**

 1. I thought the authors did a good job of describing the strengths and limitations of the study and putting this study into the context of the ANISA and SANISA trials.

*Response: Many thanks for this comment. Results obtained from our study, ANISA, and SANISA demonstrate the need for more advanced diagnostics such as the use of pathogen-specific biomarkers and metagenomics to better understand the epidemiology of neonatal sepsis in low- and middle-income countries, given the large proportion of cases lacking causal attribution following use of TAC reverse-transcription quantitative polymerase chain reaction.*

**Reference**

 1. Seale AC, Obiero CW, Jones KD, et al. Should First-line Empiric Treatment Strategies for Neonates Cover Coagulase-negative Staphylococcal Infections in Kenya? *The Pediatric infectious disease journal* 2017;36(11):1073-78. doi: 10.1097/INF.0000000000001699 [published Online First: 2017/07/22]
